# Extensive Assessment of Underlying Etiological Factors in Primary Infertile Men Reduces the Proportion of Men With Idiopathic Infertility

**DOI:** 10.3389/fendo.2021.801125

**Published:** 2021-12-24

**Authors:** Eugenio Ventimiglia, Edoardo Pozzi, Paolo Capogrosso, Luca Boeri, Massimo Alfano, Walter Cazzaniga, Rayan Matloob, Costantino Abbate, Paola Viganò, Francesco Montorsi, Andrea Salonia

**Affiliations:** ^1^ Division of Experimental Oncology/Unit of Urology, Urological Research Institute (URI), Istituto di Ricerca e Cura a Carattere Scientifico (IRCCS) Ospedale San Raffaele, Milan, Italy; ^2^ Department of Urology, University Vita-Salute San Raffaele, Milan, Italy; ^3^ Department of Urology, Istituto di Ricerca e Cura a Carattere Scientifico (IRCCS) Fondazione Ca’ Granda – Ospedale Maggiore Policlinico, University of Milan, Milan, Italy; ^4^ Infertility Unit, Unit of Obstetrics/Gynecology, Istituto di Ricerca e Cura a Carattere Scientifico (IRCCS) Ospedale San Raffaele, Milan, Italy

**Keywords:** infertility, male infertility, cause, idiopathic, risk factors

## Abstract

**Objective:**

Up to 40% of infertile men remain without a recognized cause (i.e., idiopathic infertility). We aimed to identify, categorize, and report the supposed causes of male infertility in a cohort of white-European men presenting for primary couple’s infertility, by using a thorough and extensive baseline diagnostic work-up.

**Material and Methods:**

Cross-sectional study of 1,174 primary infertile men who underwent a thorough diagnostic work-up including: detailed medical history, physical examination, hormonal assessment, genetic testing, semen analyses; semen and urine cultures; testis color Duplex US. Men without any identified causal factor were considered as idiopathic. Six different etiological categories were established, and their prevalence was estimated. Logistic regression models estimated the risk of missing causal identification.

**Results:**

A possible causal factor was identified in 928 (81%) men. Hypogonadism was the most frequent identified cause (37%), followed by varicocele (27%). Genetic abnormalities were found in 5% of patients. A causal factor was more easily identifiable for the more severe infertility cases, and azoospermic men were those less likely to be defined as idiopathic (OR and 95% CIs: 0.09; 0.04-0.20). Relative proportion of identified causes remained constant during the 10-year study period (p>0.43).

**Conclusions:**

Due to a more comprehensive and extensive diagnostic work-up, at least one underlying cause of male infertility factor in 4 out of 5 infertile men can be identified. Men with a less severe phenotype remain a clinical challenge in terms of establishing a possible etiologic factor. Further studies are needed to assess which subset of infertile men deserves a more extensive work-up.

## Introduction

Lack of both effective therapeutic strategies and identifiable underlying causes are common features in infertile men (1). Up to 60% of cases remain without a recognized cause, and are therefore referred to as idiopathic according to various series (1–3). Overall, this group of men is an interesting epidemiological cohort for several reasons. First, this sample represents an ideal cohort for studying new possible etiological factors linked to male subfertility and infertility (1–3). Second, the lack of an underlying etiologic factor may seriously limit further diagnostic work-up and, above all, possible therapeutic options (1). Third, in the context of clinical syndromes, it might anticipate future or yet occult health issues which would otherwise progress unnoticed in infertile, and therefore often young, men (4). Eventually, the lack of a clear explanation for their reproductive issue represents a factor of psychological distress in infertile men (5).

The definition of idiopathic infertility and its prevalence vary consistently according to previously published reports (2, 3), depending on the postulated possible causal factors and the baseline diagnostic work-up selected by the investigators. It was previously shown that a more accurate work-up may improve the diagnostic process increasing its accuracy during clinical evaluation of the infertile male (6, 7).

For these reasons, by using a thorough and extensive baseline diagnostic work-up, we aimed to identify, categorize, and report possible aetiologies of male factor infertility of a large homogenous cohort of white-European men presenting for primary couple’s infertility, and to report the rate of those men with an identifiable cause that would have otherwise classified as having idiopathic infertility with the standard diagnostic work-up.

## Methods

### Study Population

The analyses considered a homogenous cohort of 1,147 white-European men only belonging to primary infertile couples assessed between 2007 and 2016 at a single academic centre. Two different semen analyses were requested for every enrolled man and evaluated according to the 2010 WHO guidelines. Male factor infertility (MFI) was defined and identified as at least one impaired sperm parameter in at least two consecutive semen analyses and after a comprehensive gynaecological evaluation of the female partners. Data collection followed the principles outlined in the Declaration of Helsinki. All patients signed an informed consent form agreeing to share their own anonymous information for other future studies. The study was approved by the local ethic committee (IRCCS OSR Prot. 2014 – Pazienti Ambulatoriali).

### Diagnostic Work-Up

We performed an extensive diagnostic work-up for every included man, irrespective of the baseline infertility severity. This work-up included: detailed patient history (specifically also inquiring cryptorchidism, puberty onset, history of mumps, genital infections, urogenital trauma, previous urogenital/pelvic surgery, cigarette smoking, use of illicit drugs (e.g., marijuana, cocaine, opioids), use of anabolic steroids, symptoms of testosterone deficiency; comorbidities were scored with the Charlson Comorbidity Index (CCI), which was categorised as 0, 1, ≥2); physical examination (e.g., testicular volume, varicocele, genital tract abnormalities); hormonal assessment (including, total testosterone, FSH, LH performed in a fasting state in every case before 10 AM and repeated in order to confirm abnormal values); genetic testing (karyotype analysis, Y-chromosome microdeletions, CFTR gene mutations); semen analyses; semen and urine cultures; and, testis color duplex-US. Based on the results of the diagnostic work-up, six different etiological categories were established: 1) men with genetic abnormalities; 2) men with history of cryptorchidism (without genetic abnormalities); 3) men with genital tract obstructions (without known genetic abnormalities and cryptorchidism); 4) men with biochemical hypogonadism (defined as FSH>7.8 mU/ml and/or total testosterone <3 ng/ml and/or LH >9.4 mU/ml; without genetic abnormalities, cryptorchidism, and genital tract obstructions) (8, 9); 5) men with clinical varicocele (color duplex-US confirmed, without genetic abnormalities, cryptorchidism, genital tract obstruction, and hypogonadism); and, 6) men with other factors (either current or history of seminal tract infections, medical or physical treatment likely to affect fertility, trauma, and other iatrogenic causes) in absence of varicocele, genetic abnormalities, cryptorchidism, genital tract obstructions, and hypogonadism. Thereof, men without any identified causal factor were considered idiopathic.

We further distinguished between isolated MFI or a mixed infertility factor. MFI was defined after a comprehensive diagnostic evaluation of all the female partners.

### Statistical Analyses

Statistical analyses consisted of several steps. First, we assessed the prevalence of each specific cause in our population, assessing the proportion of idiopathic men following the proposed extensive work-up. Second, we analysed the prevalence of each specific cause over time seeking for possible time trends. Third, we evaluated the prevalence of idiopathic infertility and each specific cause according to different severity of baseline clinical presentation; for this specific purpose, we evaluated cause prevalence at different sperm concentration thresholds, under the assumption that this parameter represents a proxy of MFI severity. Eventually, logistic regression model estimated odds ratio (OR) and 95% confidence (95% CI) intervals of the idiopathic infertility, including as model covariates patient age, BMI, comorbidities, mean testicular volume, isolated MFI vs. mixed infertility factor, and azoospermia. Distribution of data was tested with the Shapiro–Wilk test. Data are presented as medians (interquartile range; IQR) or frequencies (proportions). All statistical tests were two-sided with a significance value set a 0.05.

## Results

**Table 1** details descriptive statistics of the whole cohort of patients. Median (IQR) age of the study cohort was 37 (34-41) years. Most of the included men had an isolated MFI (791, 69%) and a CCI score of 0 (1080, 94%). Men with isolated MFI did not considerably differ from men with a mixed infertility factor, except for higher sperm concentration (9 (1-35) vs. 4 (0-24) 10^6^ spermatozoa/ml, p<0.001 at Mann-Whitney test).

**Table 1:** Descriptive characteristic of the whole cohort of patients [No. 1,147].

	MFI	Mixed factor	Overall
	n = 791	n = 356	n = 1147
**Age (years)**			
Median (IQR)	37 (34-40)	37 (34-41)	37 (34-41)
**BMI (kg/m^2^)**			
Median (IQR)	25 (23-27)	25 (23-27)	25 (23-27)
**CCI - n (%)**			
0	748 (95)	332 (93)	1080 (94)
1	21 (3)	12 (3)	33 (3)
2+	22 (3)	12 (3)	34 (3)
**Mean testicular volume (Prader)**			
Median (IQR)	15 (12-20)	15 (12-20)	15 (12-20)
**Total testosterone (ng/ml)**			
Median (IQR)	4 (3-6)	5 (3-6)	4 (3-6)
**FSH (mU/mL)**			
Median (IQR)	6 (3-13)	5 (3-10)	6 (3-11)
**Sperm concentration (10^6^/ml)**			
Median (IQR)	4 (0-24)	9 (1-35)	6 (0-26)
**Varicocele - n (%)**			
No	423 (53)	191 (54)	614 (54)
Yes	368 (47)	165 (46)	533 (46)
**Cryptorchidism - n (%)**			
No	703 (89)	326 (92)	1029 (90)
Yes	88 (11)	30 (8)	118 (10)
**Karyotype abnormalities - n (%)**			
Normal	499 (93)	233 (95)	732 (94)
XXY	12 (2)	5 (2)	17 (2)
Other abnormalities	24 (4)	7 (3)	31 (4)
**CFTR - n (%)**			
Normal	788 (100)	356 (100)	1144 (100)
Mutation	3 (0)	0 (0)	3 (0)
**Y chromosome microdeletions - n (%)**			
Normal	780 (99)	355 (100)	1135 (99)
Deletion	11 (1)	1 (0)	12 (1)
**Cause of infertility - n (%)**			
Idiopathic	142 (18)	77 (22)	219 (19)
Genetic abnormalities	48 (6)	13 (4)	61 (5)
Cryptorchidism	73 (9)	25 (7)	98 (9)
Obstructive	16 (2)	5 (1)	21 (2)
Hypogonadism	295 (38)	125 (35)	420 (37)
Varicocele	199 (25)	105 (30)	304 (27)
Other	11 (1)	3 (1)	14 (1)

The study cohort is stratified according to the presence of an isolated male factor infertility (MFI) or a mixed factor (MFI + female factor).

BMI, body mass index; CCI, Charlson comorbidity index; CFTR, cystic fibrosis conductance regulator; FSH, follicle stimulating hormone.

We were able to identify and define a causal category for 928 out of 1,147 men (81%). The most common causal category was hypogonadism (420 men, 37%), whereas genetic factors were identified in 61 men (5%, **Table 1**).

**Figure 1:**
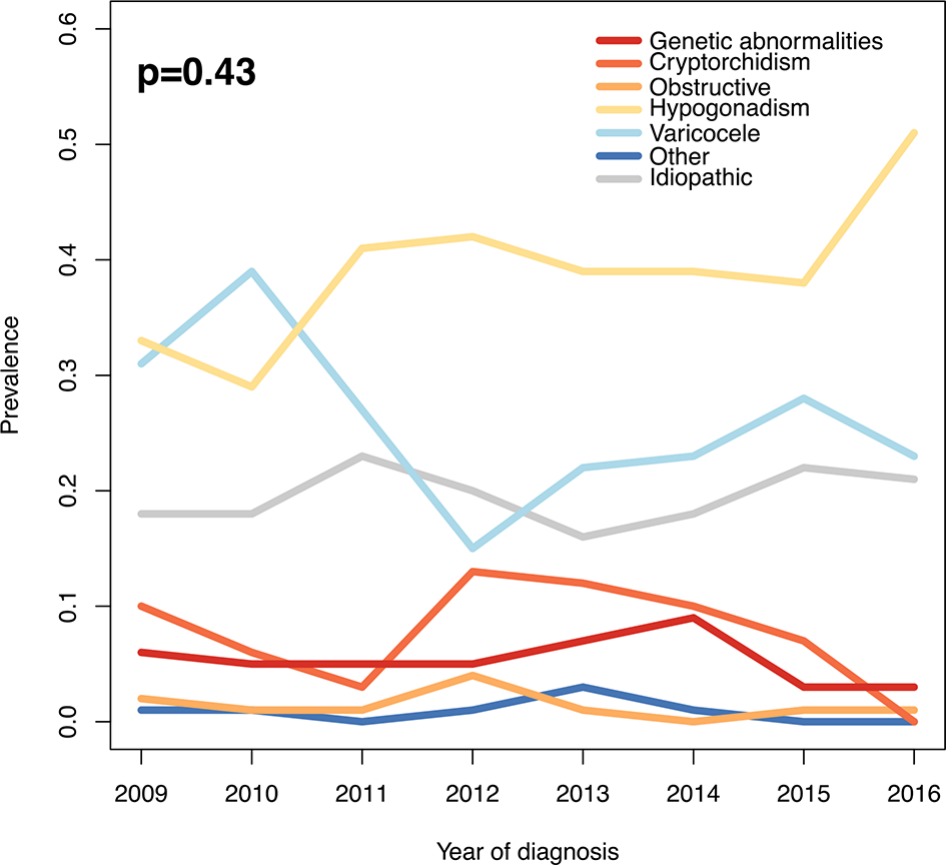
Prevalence of different causes of male factor infertility during the study period. P-value as for Chi-square test.

During the analyzed 10-year study period we found no difference in prevalent causes of MFI over time (p=0.43 as for Chi square test, **Figure 1**).

**Figure 2:**
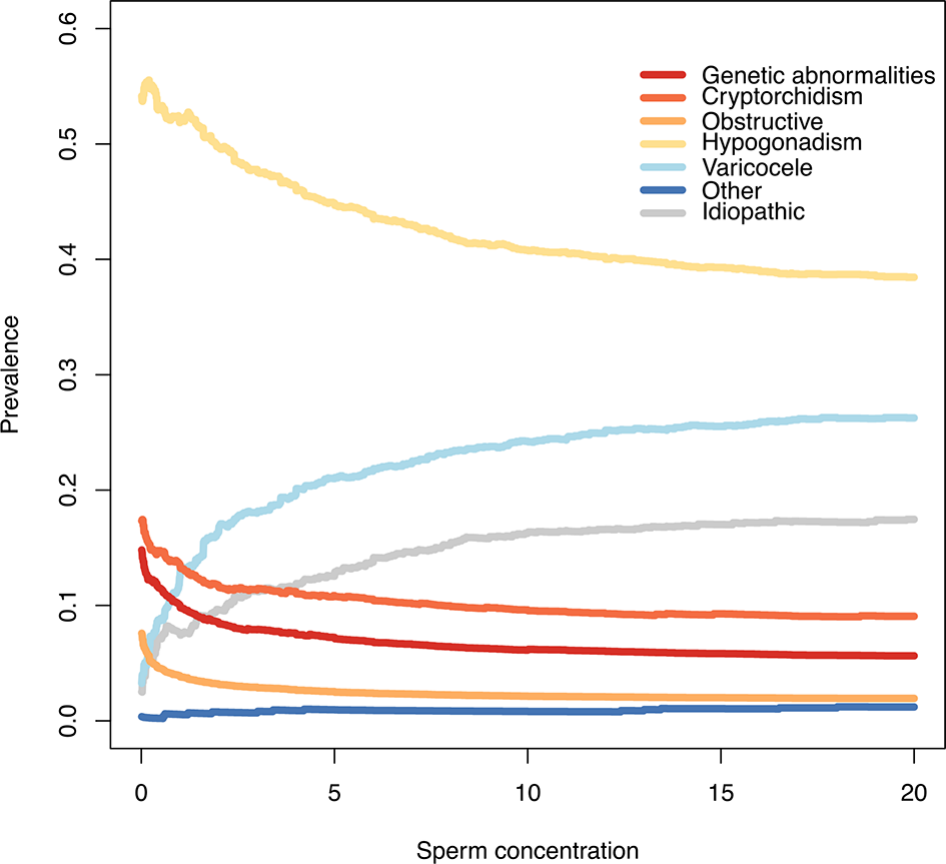
Prevalence of different causes of male factor infertility according to different sperm concentration thresholds. At each sperm concentration threshold (x axis) is shown on the y-axis the relative proportion of men with a specific causal factor (eg: if x=5, on the y-axis is shown cause prevalence of men with sperm concentration < 5 million/ml).

As shown in **Figure 2**, men with a more severe MFI were less likely to be classified as idiopathic: a lower proportion of idiopathic cases was observed in men with azoospermia compared to men with sperm concentration > 10 million spermatozoa/ml (3% vs. 34%, p<0.01 as for Chi square test).

At multivariable logistic regression analysis (**Table 2**), men with a larger testicular volume (OR: 1.07; 1.04 - 1.10) were at higher risk of having an idiopathic MFI, whereas azoospermic men (OR: 0.09; 0.04 - 0.20) had a reduced risk of missing a causal identification.

**Table 2:** Multivariable OR and 95% CI for the diagnosis of idiopathic infertility in the study cohort [No. 1,147].

	OR	95% CIs	p
**Age**			
years	1.03	1.00 - 1.05	0.05
**BMI**			
kg/m^2^	0.97	0.93 - 1.02	0.26
**CCI**			
0	1.00	(Ref.)	
1	0.97	0.38 - 2.50	0.95
2+	1.64	0.69 - 3.88	0.26
**Mean testicular volume**			
prader	1.07	1.04 - 1.10	<0.001
**Infertility factor**			
MFI	1.00	(Ref.)	
Mixed factor	1.10	0.79 - 1.52	0.58
**Azoospermia**			
no	1.00	(Ref.)	
yes	0.09	0.04 - 0.20	<0.001

BMI, body mass index; CCI, Charlson comorbidity index; MFI, male factor infertility; Mixed factor MFI + female factor.

## Discussion

In this study, we performed an extensive diagnostic work-up in 1,147 white-European men with MFI only belonging to couples complaining primary infertility in order to properly and precisely assess the possible underlying causal factors. By applying this extensive work-up, it was possible to identify a causal category for 81% of the study cohort. Moreover, men with a less severe MFI were those with the highest chance of missing a causal identification.

Uro-andrologists working in the reproductive medicine setting often face both lack of effective therapeutic options for MFI patients and a misclassification of the disease etiology (1). This has several drawbacks in terms of further diagnostic work-up and treatment (6, 7); it should be considered as well that the identification of possible causal factors might bring psychological relief to the infertile couple. Though several improvements were reported throughout the last years (10), the way we assess and treat infertile couple remains still unsatisfactory in a relevant proportion of cases.

The most comprehensive and recently available reports set the proportion of idiopathic cases between 35% and 60% (2, 3). Such wide differences depend both on selection criteria at study entry and the way causal categories were defined. Punab et al. (3) analyzed data from 1,737 men, establishing *a priori* seven causal factors (secondary hypogonadism, seminal tract obstruction, genetic causes, oncological diseases, severe sexual dysfunction, congenital uro-genital abnormalities, and acquired testicular damage) further sub-classified into absolute, severe, and plausible factors; the authors found that 40% of the study cohort was classified into the aforementioned categories. Of importance, varicocele was not considered as a causal factor. Conversely, Olesen et al. (2) established a wider pool of causes, including varicocele (13% of the analyzed cohort), limiting the idiopathic proportion of infertile men to one third of the whole cohort; the most frequently identified factor was cryptorchidism (17%). It should be noted that different selection criteria were used in the two aforementioned studies: Olesen et al. (2) selected men referred for diagnostic work-up prior to *in vitro* fertilization (IVF) or intra-cytoplasmatic sperm injection (ICSI) treatments, whereas Punab et al. (3) included infertile men with severe male factor infertility defined by total sperm count <39 million per ejaculate. Different inclusion criteria are likely to result in different prevalence of the underlying causal categories, since we clearly showed that cause-specific prevalence varies according to MFI severity. Moreover, not only inclusion criteria are likely to influence prevalence results: the selected work-up will similarly impact on study finding, since >30% of included men did not undergo genetic testing in the study by Punab et al. (3). Most of the previous efforts in better ascertaining MFI were directed towards men with the most severe clinical presentation in terms of reproductive disorders, azoospermia (11, 12). Our group previously showed that a more extensive and tailored work-up is able to reduce the misdiagnosis of hypogonadism (7) and karyotype abnormalities in infertile men (6). Similarly, we show in this study that idiopathic cases can be limited by using a more extensive work-up. For these reasons, we aimed at designing a study capable of maximizing the ascertainment of possible causal factors (by means of an extensive work-up) in a cohort of men with MFI without entry restrictions. As a consequence, this allowed us to stratify cause prevalence according to MFI severity in the widest and most accurately ever analyzed homogenous sample of white-European primary infertile men.

There is still an ongoing and longstanding epidemiological debate regarding whether specific factors should be considered as either causal or risk factors (13). For the specific purpose of our study, we defined causal categories relying on previously published reports which examined consistently possible causes or strongly related risk factors, building a hierarchical classification up. At this regards, genetic abnormalities (GA; including karyotype abnormalities, CFTR mutations known to impair fertility, and Y-chromosome microdeletions) represent one of the very few indisputable and ascertained cause of male reproduction impairment (1). For this reason, we decided to give GA the highest position in our hierarchical classification.

Men with cryptorchidism and TDS symptoms but without genetic abnormalities ranked second in this hierarchical grouping. These men share a clear condition linked to MFI which dates back to the developmental age (14), despite lacking an identifiable shared genetic background (in its non-syndromic presentation) (15). At this regard, 9% of our cohort was included in this category, being more common in men with a more severe MFI.

We decided to consider men with biochemical hypogonadism as a category on its own for several reasons. For this specific purpose, we considered previously published European Male Aging Study (EMAS) criteria for defining biochemical hypogonadism (8) implementing them with FSH values according to Barbotin et al. (9). Notably, hypogonadal men represented the largest category in our population (37%). Including men with primary, secondary, and compensated hypogonadism allowed us to intercept a wide range of conditions eventually resulting in an alteration of the hormonal milieu (16), e.g. ranging from testicular deficiency to endocrine disruptors. We previously showed that not only hypogonadism is a frequent finding in infertile men, but it represents as well a heterogeneous category amenable to be further stratified into different and well defined prognostic categories (17).

We also decided to include clinical varicocele in our causal classification. It is still debated whether varicocele represents a condition unequivocally linked to MFI, and whether it should always be treated or not in this setting (1, 18, 19). In a subgroup analyses of five randomized control trials comparing treatment to observation in men with a clinical varicocele, oligozoospermia and otherwise unexplained infertility (i.e. the way we classified varicocele in this study), it was observed that varicocele repair improved pregnancy rate and live birth rate (20).

Our study is not devoid of limitations. First, the proposed extensive work-up inevitably results in overtreatment, with inherent extra costs. Future efforts will be devoted to better tailor such an extensive work-up without losing diagnostic powers. Second, there is a plethora of emerging metabolic and environmental factors detrimentally acting on male reproductive function (10, 16, 21–25), with a close interplay with general health status (25, 26); it will be interesting to see whether these factors, not considered in our classification, will gain the scientific dignity and eventually become causal factors. Third, the lack of a control group prevented us from inquiring the strengths of causal associations. Despite these limitations, we believe that the proposed user-friendly classification can be easily implemented and reproduced, casting light in a field where the everyday clinical practice still faces several grey areas.

## Conclusions

By performing a more detailed and comprehensive diagnostic work up for men with male factor infertility, it is possible to identify at least one underlying cause of male factor infertility in 4 out of 5 of these men. In this regard, this subset of men would have been recognised as having idiopathic infertility, with standard diagnostic exams. It remains a clinical challenge to establish an identifiable aetiology among infertile men with a less severe phenotype.

## Data Availability Statement

The original contributions presented in the study are included in the article/supplementary material. Further inquiries can be directed to the corresponding author.

## Ethics Statement

The studies involving human participants were reviewed and approved by “Pazienti ambulatoriali”. The patients/participants provided their written informed consent to participate in this study.

## Author Contributions

Conception and design: EV and AS. Acquisition of data: EV, PC, LB, WC, RM, CA, and EP. Analysis and interpretation of data: EV and AS. Drafting of the manuscript: EV. Critical revision: AS, PV, MA, and FM. Statistical analysis: EV and AS. Administrative, technical, or material support: AS and FM. Supervision: AS and FM. All authors contributed to the article and approved the submitted version.

## Conflict of Interest

The authors declare that the research was conducted in the absence of any commercial or financial relationships that could be construed as a potential conflict of interest.

## Publisher’s Note

All claims expressed in this article are solely those of the authors and do not necessarily represent those of their affiliated organizations, or those of the publisher, the editors and the reviewers. Any product that may be evaluated in this article, or claim that may be made by its manufacturer, is not guaranteed or endorsed by the publisher.
